# Placental infection by SARS-CoV-2: exploring alternative entry pathways

**DOI:** 10.1080/21688370.2025.2585246

**Published:** 2025-11-30

**Authors:** Carolina Lumi Tanaka Dino, Barbara Maria Cavalli, Carolline Konzen Klein, Felipe Paes Gomes da Silva, Nicolas Henrique Borges, Ana Catharina Joaquim, Thiago Rodrigues dos Santos, Natan de Araújo, Lucas Baena Cartens, Ana Clara Simões Flórido Almeida, Seigo Nagashima, Caroline Busatta Vaz de Paula, Cleber Machado-Souza, Lucia de Noronha, Meri Bordignon Nogueira

**Affiliations:** a Center for Health Sciences, Postgraduate Program of Obstetrics, Gynecology, and Women’s Health, Federal University of Paraná – UFPR, Curitiba, Brazil; b School of Medicine and Life Sciences, Pontifical Catholic University of Paraná – PUCPR, Curitiba, Brazil; c Center for Health Sciences, Federal University of Paraná – UFPR, Curitiba, Brazil; d Faculdades Pequeno Príncipe, Postgraduate Program of Biotechnology Applied to Child and Adolescent Health, Instituto de Pesquisa Pelé Pequeno Príncipe, Curitiba, Brazil; e School of Medicine and Life Sciences, Postgraduate Program of Health Sciences, Pontifical Catholic University of Paraná – PUCPR, Curitiba, Brazil

**Keywords:** viral infection, maternal–fetal interface, placenta, viral entry, Endocytosis

## Abstract

The placenta possesses several structural and immunological barriers against viral infections, the SARS-CoV-2 detection in placental tissues has raised concerns regarding possible alternative viral entry mechanisms beyond the canonical ACE2/TMPRSS2-mediated pathway. In this context, the present study evaluated the immunohistochemical expression patterns of ADAM17, Cathepsin L, Clathrin, ACE-2, Furin, NRP-1, and TMPRSS2—molecules involved in SARS-CoV-2 placental entry pathways – as well as the detection of viral RNA by RT-qPCR in paraffin-embedded samples. The study included 75 paraffin-embedded placental samples (decidua and villi) collected after spontaneous placental delivery at birth from patients who tested positive for COVID-19 (COVID-19 Group), and 19 paraffin-embedded control placental samples collected prior to the COVID-19 pandemic (NON-COVID-19 Group). A statistically significant reduction in NRP-1 expression was observed in the COVID-19 group decidua (*p* < 0.001), including in RT-qPCR – positive samples (*p* = 0.001), regardless of comorbidities or underlying conditions. A statistically significant reduction in Clathrin expression was also found in the decidual samples of the COVID-19 group and in RT-qPCR – positive samples (*p* = 0.05and 0.013, respectively), while Cathepsin L expression was significantly increased in the placental villi of the COVID-19 group (*p* < 0.001) and in RT-qPCR – positive samples (*p* = 0.005). These findings may contribute to a better understanding of the mechanisms underlying SARS-CoV-2 interaction with the placenta, possibly through auxiliary and/or endocytic entry pathways, and may support future investigations into the impact of these alterations in the context of maternal SARS-CoV-2 infection.

## Introduction

The placenta is an essential interface between maternal and fetal circulations for the exchange of nutrients and metabolic waste products.^1^ Its highly complex and intricate structure consists of fetal and maternal derived components. Both physical and immunological mechanisms are utilized by this organ to inhibit infection.^2^

The decidua in the maternal compartment plays a vital role as an immunological barrier in the mother-fetus interface.^3^ Also, the maternal blood circulates through the placental intervillous space, thereby allowing exchange of vital substances required for fetal homeostasis. The fetal component, chorionic villi, projects into this space, enlarging the surface of exchange and maximizing the transfer of vital components for fetal development.^4^

Apart from being a site for the exchange of substances, the placenta also serves as a shield against infection by infectious agents, utilizing several mechanisms that reduce the incidence of infections. Some of these mechanisms include physical separation of maternal and fetal circulatory systems, special immunological characteristics, and selective transport mechanisms.^5^ Despite such protection mechanisms, some viruses, like SARS-CoV-2, have the capability of infecting placental cells, an aspect that generates concern regarding possible effects on placental function and maternal-fetal well-being.^4,6–8^

The SARS-CoV-2 infection mostly occurs through viral attachment to the angiotensin-converting enzyme 2 (ACE-2) receptor, which induces the expression of transmembrane serine protease type II (TMPRSS2). Since the receptor is identified to be widely expressed in numerous human tissues, they are targeted by the virus.^7,9^ Stimulation of a disintegrin and metalloprotease 17 (ADAM17) is also seen to play a role in exacerbating COVID-19 as it takes part in enhancing inflammatory responses via shedding of inflammatory cytokines. ADAM17 also facilitates dysregulation of ACE-2, which exerts a profound impact on the cardiovascular system.^10^

**Figure 1.**
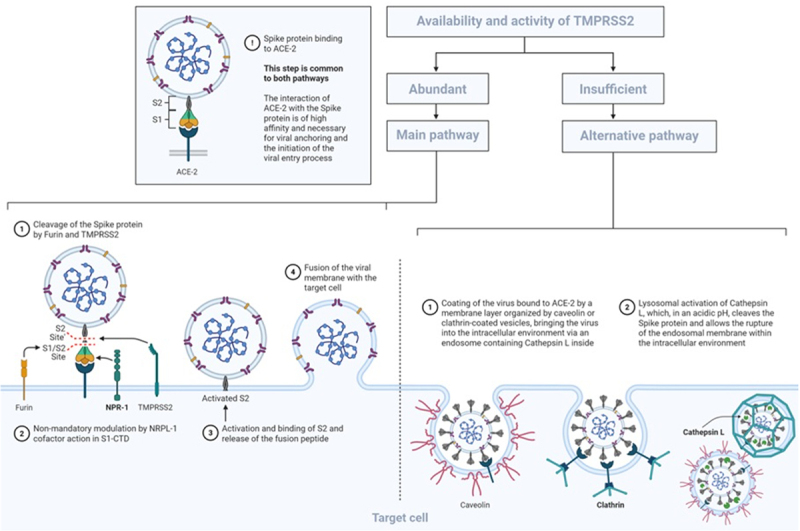
SARS-CoV-2 entry pathways.

Recent literature demonstrates the placental expression of ACE-2 to be quite low, especially during the third trimester, hence the concern regarding the potential presence of other infection mechanisms.^11^ Among the postulated mechanisms, neuropilin-1 (NRP-1) has been put forth as another receptor that could serve as a facilitator of viral entry into host cells. NRP-1 binds to the S1 subunit of the viral spike protein, triggering conformational changes that are permissive for proteolytic cleavage between the S1 and S2 domains by the transmembrane endoprotease furin. This activation of the S2 subdomain promotes the fusion between the viral and host cell membranes, launching infection.^6,7,12^ A second potential viral entry pathway involves clathrin-mediated endocytosis, a cytosol protein.^13^ In this second entry pathway, the virus attaches to the receptor and is then taken in through endocytosis. The S2 site is cleaved by cathepsins, and particularly, L-cathepsin plays a crucial role as a protease in protein degradation within late endosomes. This action promotes the release of the viral genome into the cytoplasm, thus enabling cellular infection^14^ (Figure 1).

Knowledge of these alternate infection routes is necessary not just to clarify mechanisms of placental infection but also to determine the potential, albeit infrequent, incidence of vertical transmission of the virus. Additional research can yield useful data to aid in the development of prevention and treatment strategies for SARS-CoV-2 infection in pregnancy, thus enhancing safeguarding against maternal-fetal health.

## Materials and methods

### Ethical approval

This study was approved by the Research Ethics Committee under the CAAE number: 35,129,820.60000.0096. The authors ensure that all procedures were conducted in accordance with ethical guidelines, current regulations, and applicable data security protocols. The collection of biological material was authorized by the families, who provided formal consent by signing the informed consent form (ICF).

### Samples

#### COVID-19 group (n = 76)

Placental samples were collected between June 2020 and August 2021 from pregnant patients over 14 years of age who were diagnosed with SARS-CoV-2 at the time of hospitalization, regardless of gestational age, symptom presence, or comorbidities. Patients were admitted to the Hospital de Clínicas Complex – Federal University of Paraná (CHC-UFPR) or the Hospital Nossa Senhora das Graças (HNSG), both located in Curitiba, Paraná, Brazil.

COVID-19 testing was performed using nasopharyngeal swabs collected during hospitalization, with viral detection by real-time polymerase chain reaction (RT-qPCR). The detection kits used included XGEN MASTER COVID-19, SARS-CoV-2 IBMP Molecular Kit, and Xpert Xpress, with positive results confirming SARS-CoV-2 infection.

Placentas were obtained following the natural placental expulsion process during delivery. Criteria for submission to histopathological examination included maternal or fetal conditions previously diagnosed during prenatal care, severe abnormalities observed during delivery, or multiple pregnancies, in accordance with routine protocols at both institutions. The positive RT-qPCR test for SARS-CoV-2 in the nasopharyngeal swab was considered an abnormal maternal condition and a criterion for histopathological evaluation of the placenta.

During the study, one sample was excluded due to the revocation of the informed consent form (ICF) by the patient or their legal guardian, resulting in a total of 75 samples analyzed.

#### NON-COVID-19 group (n = 19)

Formalin-fixed, paraffin-embedded (FFPE) placental tissue samples from CHC-UFPR were collected between 2016 and 2018. The analysis protocol for these placentas was similar to that used for the COVID-19 Group. Tissue samples were tested using immunohistochemistry techniques, with negative results for congenital diseases and the TORCHZ pathogens (toxoplasmosis, rubella, cytomegalovirus, herpes simplex virus types 1 and 2, and Zika virus).

### Clinical information

#### COVID-19 group

Clinical and laboratory data were obtained from medical records during hospitalization. The collected information included maternal age, presence or absence of comorbidities, parity, gestational age at delivery, type of delivery, newborn birth weight, APGAR score, reason for hospitalization, severity of maternal illness, gestational trimester of COVID-19 infection, length of hospitalization, interval between hospitalization and delivery, intrauterine fetal demise, neonatal death, newborn symptoms or positive test for SARS-CoV-2, and the presence of placental histopathological abnormalities.

Unfortunately, complete perinatal outcome data (e.g., APGAR scores, birth weight percentiles, intrauterine infection rates) were not available for all cases in our cohort. This limitation is acknowledged and we suggest that future prospective studies evaluate the relationship between placental expression of SARS-CoV-2 entry-related proteins and newborn outcomes.

Additionally, information regarding the presence or absence of symptoms typically associated with COVID-19 was collected (temperature above 38°C, dyspnea, cough, myalgia, nausea and vomiting, diarrhea, headache, anosmia, and dysgeusia). Maternal disease severity was categorized as follows: asymptomatic/mild symptoms; moderate severity requiring hospitalization in a general ward; severe cases with critical organ dysfunction requiring intensive care unit (ICU) admission.

#### NON-COVID-19 group

Clinical and laboratory data were obtained from medical records with information similar to that collected for the COVID-19 Group.

### Histopathological and Qualitative morphological analysis

Representative placental samples from the COVID-19 Group were fixed in 10% buffered formalin and embedded in paraffin (FFPE), undergoing routine processing for histopathological assessment. The placental samples in the NON-COVID-19 Group were already embedded in paraffin blocks.

The qualitative morphological analysis of placentas from both groups was conducted following the Amsterdam Placental Workshop Group Consensus Statement.^11^ Prior to analysis, the paraffin-embedded blocks were renamed and blindly examined by two pathologists. Findings were classified as “present” or “absent,” and the extent and intensity of specific alterations were graded according to the recommendations outlined in the referenced protocol.

### Immunohistochemical analysis

The immunohistochemistry assay was preceded by the preparation of multi-sample paraffin tissue blocks, known as Tissue Microarrays (TMA). Representative placental areas were pre-marked and identified. Subsequently, four cylindrical fragments, two from the decidua and two from the villous tissue – each measuring 0.8 cm in diameter, were extracted from the original donor blocks and compiled into new TMA blocks.

The immunohistochemistry technique was applied to assess the immunoexpression of ADAM17, ACE-2, Clathrin, Furin, L-Cathepsin, NRP-1, and TMPRSS2, as detailed in Table 1.

**Table 1.** Data on the antibodies used in the study.

ANTIBODY	BRAND	CLONE/CODE	DILUTION	MONO/POLYCLONAL	*HOST*
Anti-ADAM17	Clonality	AB-39163	1:100	Policlonal	*Rabbit*
Anti-L-Cathepsin	Elabscience	EC9134	1:200	Policlonal	*Rabbit*
Anti-Clathrin	Abcam	Ab172958	1:200	Monoclonal	*Mouse*
Anti-ACE-2	Elabscience	EAB-12224	1:100	Policlonal	*Mouse*
Anti-Furin	Elabscience	EAB-61709	1:200	Policlonal	*Rabbit*
Anti-NRP-1	Clonality	AB-25998	1:100	Policlonal	*Rabbit*
Anti-TMPRSS2	BioSB	BSB-136	1:100	Monoclonal	*Mouse*

The technical protocol recommended overnight incubation of the primary antibodies. The secondary polymer (Mouse/Rabbit PolyDetector DAB HRP Brown, BioSB, BSB0205, Santa Barbara, CA,USA) was applied to the material at room temperature. The reaction was visualized by adding the 2,3-diaminobenzidine + hydrogen peroxide substrate complex. Positive and negative controls were used to validate the reactions.

The immunolabeled slides were digitized using the Axio Scan.Z1 scanner (ZEISS, Jena, Germany) and processed for the generation of 50 HPF (high power fields) for the COVID-19 group and 50 HPF for the NON-COVID-19 group, using the ZEN Blue Edition software (ZEISS, Jena, Germany). Analyses were conducted in a blinded manner by an independent observer. Immunoexpression areas were quantified using Image Pro-Plus 4.5 software (Media Cybernetics, Rockville, MD, USA) and subsequently converted into percentages. The data were then subjected to statistical analysis.

### Viral extraction and RT-qPCR

For the RT-qPCR analysis of paraffin-embedded placental samples from the COVID-19 and NON-COVID-19 groups, small original block fragments were aseptically collected using a dedicated scalpel for each sample. The area of interest was pre-marked microscopically by a pathologist. The fragments were deparaffinized using 1 mL of xylene and subjected to viral RNA extraction using the RNeasy FFPE Kit Qiagen®, following the manufacturer’s instructions.

RT-qPCR was performed using the SARS-CoV-2 Allplex® Assay Kit (Seegene Inc., Korea), enabling the simultaneous amplification and detection of the E, RdRP, and N genes. An endogenous gene was used as an internal control (IC) to monitor nucleic acid extraction and detect potential PCR inhibition. RT-qPCR reactions were conducted using the Applied Biosystems 7500 Real-Time PCR System (Applied Biosystems™, California, USA), and analyses were performed with the system’s proprietary software, following the manufacturers’ protocol guidelines.

### Statistical analysis

Means, standard deviations, medians, maximum and minimum values, frequencies, and percentages were used to describe the results. Nominal variables were expressed as absolute values and frequencies and analyzed using the chi-square test or Fisher’s exact test, as appropriate. The normality of quantitative variables was assessed using the Shapiro – Wilk test. Normally distributed variables were compared using Student’s t-test, while non-normally distributed variables were analyzed using the Mann – Whitney test. The Kruskal – Wallis test was applied to compare quantitative variables among multiple groups. A statistical significance level of *p* ≤ 0.05 was adopted.

Given the number of comparisons performed, p-values were also adjusted using the Bonferroni correction, applied only to the set of analyses for which multiple related comparisons were conducted. These analysis were performed using JMP Pro® 14.0.0 software (SAS Institute, Cary, NC, USA). Results remained statistically significant only if the Bonferroni-adjusted p-value was < 0.05. A complete table containing both the original and Bonferroni-adjusted p-values is provided in the Supplementary Material.

## Results

The demographic, clinical, and histopathological analyses of the groups are summarized in Table 2.

**Table 2.** Comparison between the NON-COVID-19 and COVID-19 groups regarding clinical and pathological findings.

Data	NON-COVID-19 Group (*n*=11)	COVID-19 Group (*n*=75)	*p* value
Maternal age (years)^1^	28.3 ± 8.42 (18–42)	30.6 ± 5.99 (14–42)	0.400
Gestational age (weeks)^1^	34 ± 4.87 (23.6–39)	36.3 ± 3.68 (23.9–41)	0.157
Reason for hospitalization	–	COVID-(40) 53.3%	
	-	Delivery (35) 46.7%	
Disease severity (COVID-19)^2^	Mild or asymptomatic -	Mild or asymptomatic (41) 54.7%	
	-	Moderate (13) 17.3%	
	-	Severe (21) 28%	
Trimester of SARS-CoV-2 infection^2^	–	First (4) 5,33%	
	-	Second (7) 9,33%	
	-	Third (64) 85,33%	
Mode of delivery^2*^	Cesarean section (5) 45.45%	Cesarean section (55) 72.4%	0.084
	Vaginal (6) 54.55%	Vaginal (21) 27.6%	
Comorbidities^2^	Yes (2) 18.2%	Yes (40) 53.3%	**0.05**
	No (9) 81.8%	No (35) 46.7%	
Hypertensive Disorders of Pregnancy (HDP)^2^	Yes (0) 0%	Sim (8) 10.7%	0.589
	No (11) 100%	No (67) 89.3%	
Diabetes^2^	Yes (1) 9.1%	Yes (10) 13.3%	1.00
	No (10) 90.9%	No (65) 86.7%	
Hypertension^2^	Yes (1) 9.1%%	Yes (2) 2.7%	0.340
	No (10)90.9%%	No (73) 97.3%	
Obesity^2^	Yes (0) 0%	Yes (10) 13.3%	0.348
	No (11) 100%	No (65)86.7%	
HIV positive^2^	Yes (0) 0%	Yes (2) 2.70%	1.00
	No (11) 100%	No (73) 97.30%	
Syphilis positive^2^	Yes (0) 0%	Yes (2) 2.70%	1.00
	No (11) 100%	No (73) 97.30%	
Smoking^2^	Yes (1) 9.09%	Yes (4) 12.3%	0.504
	No (10) 90.91%	No (71) 87.7%	

^1^Quantitative variables are presented as mean ± standard deviation (minimum–maximum). Student’s t-test for independent samples was used. ^2^Qualitative variables are presented as absolute values (n) and relative frequencies (%). Fisher’s exact test was applied. p-values < 0.05 were considered statistically significant.

*The total number exceeds 75 in the COVID-19 group due to a twin pregnancy among the participants.

The results indicate that pregnant women in the NON-COVID-19 group had a mean age of 28.3 years (18–42), whereas those in the COVID-19 group had a higher mean age of 30.6 years (14–42) (*p* = 0.400). The mean gestational age was comparable between groups, being 34 weeks (23–39) in the NON-COVID-19 group and 36.3 weeks (24–41) in the COVID-19 group. In the COVID-19 group, hospitalization was primarily due to SARS-CoV-2 infection (53.3%), and most patients presented with mild or asymptomatic disease (54.7%). Cesarean delivery was less frequent in the NON-COVID-19 group (45.45%) compared to the COVID-19 group (72.4%).

Comorbidities were reported in 18.2% of women in the NON-COVID-19 group, whereas 53.3% of women in the COVID-19 group had at least one comorbidity, although this difference did not reach statistical significance (*p* = 0.05).

Hypertensive disorders of pregnancy (HDP) were not observed in the NON-COVID-19 group, while 10.7% of participants in the COVID-19 group presented with this condition (*p* = 0.589). Diabetes was slightly more prevalent in the NON-COVID-19 group (9.1%) compared to 13.3% in the COVID-19 group (*p* = 1.00). Hypertension was identified in 9.1% of the NON-COVID-19 group, compared to 2.7% in the COVID-19 group (*p* = 0.340).

No cases of obesity were reported in the NON-COVID-19 group, whereas 13.3% of women in the COVID-19 group were classified as obese (*p* = 0.348). None of the comparisons regarding comorbidities between the groups reached statistical significance.

HIV and syphilis infections were absent in the NON-COVID-19 group but were detected in 2.7% of participants in the COVID-19 group (*p* = 1.00 for both comparisons). Smoking was slightly less frequent among women in the NON-COVID-19 group (9.09%) compared to 12.3% in the COVID-19 group, with no significant difference between groups (*p* = 0.504).

The demographic, clinical, and pathological analyses related to the fetuses and placentas of the respective participants, divided between groups, are summarized in Table 3.

**Table 3.** Comparison between the NON-COVID-19 and COVID-19 groups regarding demographic, clinical, and pathological findings related to the fetuses and placentas of the respective maternal participants.

Data	NON-COVID-19 Group (*n*=11)	COVID-19 Group (*n*=75) *	*p* value
Fetal sex^2^	Female (6)	Female (33) 51.5%	1
	Male (5) 45.5%	Male (31) 48.4%	
Fetal weight (g)1	2656 ± 1003	2752 ± 722	0.708
Fetal death^2^	YES (0) 00.0%	YES (2) 2.7%	1
	NO (11) 100%	NO (73) 97.3%	
Presence of COVID-19 symptoms in the fetus	–	Sintomatic (4) 5.4%	
	-	Assintomatic (70) 94.6%	
Placental weight (g)^1^	527 ± 152	438 ± 126	**0.036**
Placental pathological alterations^2^	Yes (9) 81.81%	Yes (57) 85.1%	0.675
	No (2) 18.2%	No (10) 14.9%	

^1^Quantitative variables are presented as mean ± standard deviation. Student’s t-test for ^1^Quantitative variables are presented as mean ± standard deviation. Student’s t-test for independent samples was applied.

^2^Qualitative variables are presented as absolute numbers (n) and relative frequencies (%). Fisher’s exact test was used. p-values < 0.05 were considered statistically significant. *Data missing for one fetus in the COVID-19 group. *Data missing for eight participants in the COVID-19 group.

When comparing fetal and placental outcomes between groups, the distribution of fetal sex was similar, with male fetuses comprising 45.5% of cases in the NON-COVID-19 group and 48.4% in the COVID-19 group, showing no statistically significant difference (*p* = 1.000). The mean fetal weight was 2656 g in the NON-COVID-19 group and 2752 g in the COVID-19 group (*p* = 0.708).

Fetal death was not observed in the NON-COVID-19 group, while it occurred in 2.7% of cases in the COVID-19 group (*p* = 1.000). The presence of COVID-19-related symptoms in fetuses was reported in 5.4% of cases in the COVID-19 group, whereas the vast majority (94.6%) remained asymptomatic.

A statistically significant difference was observed in placental weight, with a higher mean in the NON-COVID-19 group (527 g) compared to the COVID-19 group (438 g) (*p* = 0.036).

Macroscopic placental alterations were present in 81.8% of the NON-COVID-19 group and in 85.1% of the COVID-19 group, with no statistically significant difference between the groups (*p* = 0.675).

The comparison of tissue immunoexpression of the markers ADAM17, Clathrin, ACE-2, Furin, Cathepsin-L, NRP-1, and TMPRSS2 in both placental compartments (decidua and villi) between the NON-COVID-19 and COVID-19 groups is presented in Table 4, and Figures 2a and 2b.

**Figure 2.**
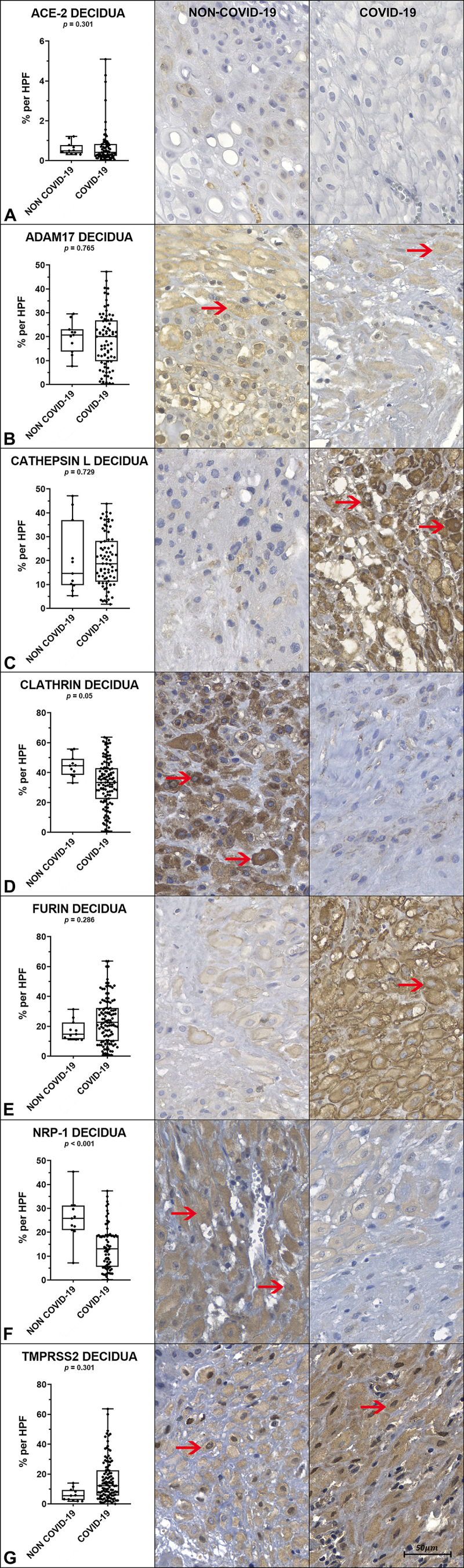
a. Comparison of tissue immunoexpression of the decidual surface between the NON-COVID-19 and COVID-19 groups, with representative image examples corresponding to each group (Image on the left).

The graphs, followed by representative images of each group, correspond to comparisons of the positively immunostained areas between them. Graphs A, B, C. D E, F, G shows tissue immunostaining values for ACE-2, ADAM17, Cathepsin L, Clathrin, Furin, NRP-1 and TMPRSS2, respectively. Red arrows indicate decidual tissue regions and cells with positive immunostaining for the respective antibodies. *p* < 0.05 indicates statistical significance. Non-parametric Mann-Whitney U test. Scale bar represents 50 μm.

**Figure 2.**
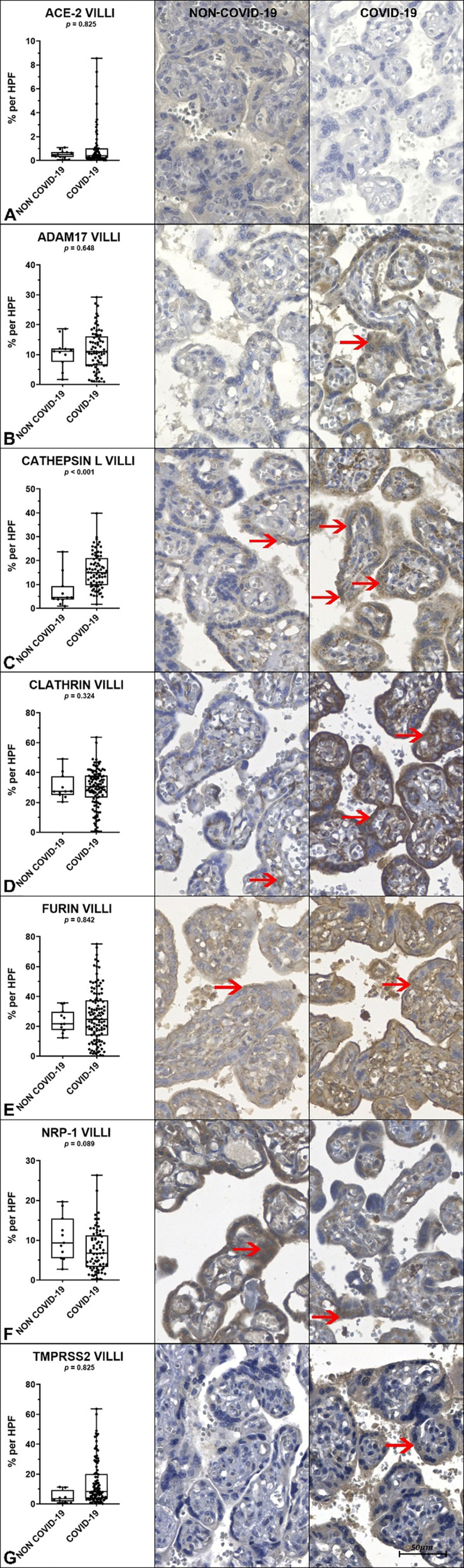
b. Comparison of tissue immunoexpression of the villous surface between the NON-COVID-19 and COVID-19 and groups, with representative image examples corresponding to each group (Image on the right).

The graphs, followed by representative images of each group, correspond to comparisons of the positively immunostained areas between them. Graphs A, B, C. D E, F, G shows tissue immunostaining values for ACE-2, ADAM17, Cathepsin L, Clathrin, Furin, NRP-1 and TMPRSS2, respectively. Red arrows indicate villous tissue regions and cells with positive immunostaining for the respective antibodies. *p* < 0.05 indicates statistical significance. Non-parametric Mann-Whitney U test. Scale bar represents 50 μm.

**Table 4.** Comparison of tissue immunoexpression in both placental compartments (decidua and villi) between the NON-COVID-19 and COVID-19 groups.

Immunomarkers	DECIDUA		*p*-value	VILLI		*p*-value
	NON-COVID-19 Group Median/(IQR)*	COVID-19 Group Median/(IQR)*		NON-COVID-19 Group Median/(IQR)*	COVID-19 Group Median/(IQR)*	
% ACE-2	0.49 (0.35)	0.41 (0.59)	0.301	0.49 (0.36)	0.41 (0.81)	0.825
% ADAM17	19.7/(6.57)**	18.6/(11.4)**	0.765	10.6 (5.05)**	11.6 (6.67)**	0.648
% Cathepsin L	14.6/(19.0)	18.7/(16.4)	0.729	4.55/(4.0)	15.0/(10.8)	**<0.001**
% Clathrin	44.5/(8.0)	36.0/(16.1)	**0.050**	30.5/(7.9)	31.7/(11.0)	0.324
% Furin	14.7 (8.18)	21.4/(20.0)	0.286	21.6 (10.0)	23.4 (22.2)	0.842
% NRP-1	25.8 (8.73)	13.1 (13.1)	**< 0,001**	9.38(7.70)	6.73 (7.63)	0.089
% TMPRSS2	5.59 (6.09)	8.38 (11)	0.078	3.43 (4.09)	5.35 (5.33)	0.259

*IQR = Interquartile range.

**Standard deviation.

p-values < 0.05 indicate statistical significance.

Non-parametric Mann-Whitney U test.

The results indicate statistically significant differences in the expression of specific immunomarkers between the NON-COVID-19 and COVID-19 groups. In the placental villi, Cathepsin L expression was significantly lower in the NON-COVID-19 group compared to the COVID-19 group (*p* < 0.001). In the decidua, Clathrin showed higher expression in the NON-COVID-19 group, with a p-value at the threshold of significance (*p* = 0.050). Additionally, NRP-1 expression in the decidua was significantly higher in the NON-COVID-19 group than in the COVID-19 group (*p* < 0.001).

No statistically significant differences were observed between groups in the expression of ACE-2, ADAM17, Furin, or TMPRSS2 in either the decidua or the villi. Similarly, Clathrin expression in the villi and NRP-1 expression in the villi did not differ significantly between groups.

**Figure 3.**
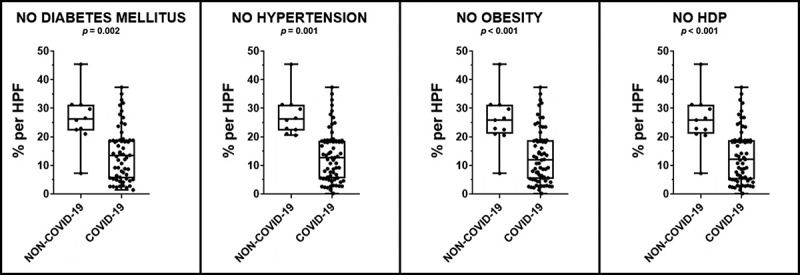
Graphs comparing the percentage of NRP-1 tissue immunoexpression in placental decidua between the NON-COVID-19 and COVID-19 groups, in relation to the absence of comorbidities and underlying conditions.

The following results (Table 5 and Figure 3) present the comparison between clinical data and NRP-1 tissue expression in the decidua from the NON-COVID-19 and COVID-19 groups, considering only pregnant women without comorbidities or preexisting conditions, such as diabetes mellitus, hypertension, obesity, or hypertensive disorders of pregnancy (HDP). This analysis aimed to determine whether the reduction in NRP-1 expression is directly induced by SARS-CoV-2 infection or influenced by underlying health conditions. Notably, even in the absence of such comorbidities, NRP-1 expression was significantly lower in the COVID-19 group.

**Table 5.** Comparison of the percentage of NRP-1 tissue immunoexpression per placental decidua between the COVID-19 and NON-COVID-19 groups, in relation to the absence of comorbidities and underlying conditions.

NO UNDERLYING DISEASES	NON-COVID-19 Group Median/(IQR)**	COVID-19 Group Median/(IQR)**	*p*-value
No Diabetes Mellitus (*n* = 9/61) *	26.2 (8.27)	13.5 (13.1)	**0.002**
No hypertension (*n*= 9/69) *	26.2(8.27)	12.7 (13.0)	**0.001**
No obesity (*n* = 11/63) *	25.8 (8.73)	12.1 (13.0)	**< 0.001**
No HDP (*n* = 11/63) *	25.8 (8.73)	12.7 (13.2)	**< 0.001**

*n= NON-COVID-19 Group/COVID-19 Group.

**IQR = Interquartile rang.

*p*-values < 0.05 indicate statistical significance.

Non-parametric Mann-Whitney U test.

Based on the RT-qPCR analysis performed on paraffin-embedded placental samples from the COVID-19 group, 11 decidual samples (SARS-CoV-2 RNA Positive Group – Decidual) and 8 villi samples (SARS-CoV-2 RNA Positive Group – Villi) were identified as positive for the presence of SARS-CoV-2 RNA. When comparing the tissue immunoexpression of the studied markers in SARS-CoV-2-positive decidual samples from the COVID-19 group, a statistically significant difference was observed for the NRP-1 receptor, which exhibited reduced tissue expression, and for Clathrin, which showed increased expression compared to the NON-COVID-19 group. Additionally, when comparing the 8 villous placental samples positive for SARS-CoV-2 in the COVID-19 group with those in the NON-COVID-19 group, a significantly increased expression of Cathepsin-L was observed in the COVID-19 group. In summary, the results from the comparison of tissue immunoexpression of the studied markers between the NON-COVID-19 Group and the SARS-CoV-2 RNA Positive Group (in both decidua and villi) closely reflect the findings previously observed in the comparison between the COVID-19 Group and the NON-COVID-19 Group (Table 6).

**Table 6.** Comparison of tissue immunoexpression of markers between the SARS-CoV-2 rna positive group (decidual and villous) and NON-COVID-19 groups in placental samples positive for SARS-CoV-2.

Immunomarkers	DECIDUA		*p*-value	VILLI		*p*-value
	SARS-CoV-2 RNA Positive Group (*n* = 11) Median/(IQR)*	NON-COVID-19 Group (*n* = 11) Median/(IQR)*		SARS-CoV-2 RNA Positive Group (*n* = 8) Median/(IQR)*	NON-COVID-19 Group (*n* = 11) Mediana/(IQR)*	
% ACE-2	0.32/(0.6)	0.49/(0.35)	0.401	0.292/(0.613)	0.498/(0.365)	0.596
% ADAM17	11.9/(11.8)	20.8/(6.95)	0.151	8.17/(12)	11.1/(3.23)	0.840
% Cathepsin L	12.8/(5.21)	14.6/(19.0)	0.332	14.4/(12.5)	4.5/(4)	**0.002**
% Clathrin	38.5/(15)	44.5/(8.0)	**0.034**	36/(14.8)	27.4/(7.9)	0.596
% Furin	10.2/(16,1)	14.7/(8.18)	0.270	17.6/(37)	21.6/(10)	0.657
% NRP-1	12.7/(13)	25.8/(8.73)	**< 0.001**	13/(4.38)	9.38/(7.7)	0.724
% TMPRSS 2	5.40/(10.9)	5.59/(6.09)	0.898	6.16/(10.4)	3.43/(4.09)	0.281

*IQR = Interquartile rang.

*p*-values < 0.05 indicate statistical significance.

Non-parametric Mann-Whitney U test.

## Discussion

For its entry into host cells, SARS-CoV-2 utilizes ACE-2 predominantly as a receptor, assisted by the TMPRSS2 protease.^15^ Recent research, however, presented an intriguing finding: downregulation of the classical proteins, namely ACE-2, in placental tissues in pregnancy, especially in the third trimester.^11,16^ This is a noteworthy observation and brings to question potential alternative mechanisms for placental infection and their relevance to the possible vertical transmission of the virus.

In the current research, the comparison of immunoexpression levels of ACE-2 and TMPRSS2 proteins revealed that there were no differences with statistical significance between decidua and placental villi samples of the groups being examined. The result indicates that the expression patterns of these proteins are comparable in various compartments of the placenta. In addition, this similarity was also seen in viral RNA analysis because no differences of note were achieved among NON-COVID-19 Group samples and those found positive for SARS-CoV-2 in the COVID-19 Group. Of note is the fact that 84.51% of placentas examined were from the third trimester, and the lack of differences between the two groups is also consistent with data from the available literature. The findings validate the hypothesis that the placenta has an invariant expression profile of these proteins irrespective of maternal infection.

Nonetheless, we must consider the possibility of other routes of viral infection in the placenta. One such pathway is mediated through NRP-1, which is a membrane-bound protein acting as a surface receptor and interacts with various protein families.^17^ Experiments suggest that NRP-1 could function as another pathway for SARS-CoV-2 cell entry as an alternative to ACE-2.^18,19^ Experiments with autopsy tissues have shown that NRP-1 is expressed abundantly in the olfactory epithelium, in contrast to ACE-2, which has negligible or no expression. The observation implies that NRP-1 could play a role in viral entry facilitation, with the possibility of allowing dissemination to the central nervous system via the olfactory epithelium.^18^

The measurement of NRP-1 immunoexpression in the decidua identified a statistically significant decrease of the protein in the COVID-19 group compared to the NON-COVID-19 group (*p* < 0.001). The same finding was noticed in SARS-CoV-2 RNA-positive samples of the COVID-19 cohort, where there was a noticeable reduction of NRP-1 expression in decidua compared to the NON-COVID-19 cohort. This decrease may be due to the use of NRP-1 as a secondary entry point for the virus or as an alternative pathway for SARS-CoV-2 infection in placental cells. NRP-1 plays a role as a cofactor in viral infection, where it increases the infectivity of SARS-CoV-2 via interaction with the C-terminal part of the S1 domain of the Spike protein, promoting the fusion of the virus with the cellular membrane.^20^ The downregulated expression of NRP-1 seen in the decidua can, therefore, indicate a cellular response to heightened viral interactions, and point toward the possible depletion of this receptor as a result of its binding with the virus.

In addition, the comparison of results between the COVID-19 Group and the NON-COVID-19 Group, taking into consideration the absence of comorbidities, revealed a decreased NRP-1 tissue immunoexpression in the former. This result indicates that the decrease in NRP-1 can be attributed to the virus depleting this receptor, rather than due to inflammatory processes prior to infection, since the existence of comorbid conditions did not influence the expression levels of this receptor.

Following the proteolytic cleavage of the Spike glycoprotein in its S1 and S2 domains through the action of proteases TMPRSS2 and furin, the S2 domain becomes activated and facilitates the direct fusion of the viral membrane with the host cell membrane.^21^ Nevertheless, SARS-CoV-2 is able to be internalized via the process of endocytosis, with the viral particle remaining intact. In this scenario, membrane fusion is achieved at the inner side of the endosomal membrane, with the viral RNA being released into the cytosol and the subsequent replication of the viral genome being possible.^22^

In the course of this research, immunoexpression of clathrin – a ubiquitously expressed intracellular protein in the placenta with a potential role in the endocytic pathway – was examined. There was significant reduction in expression of clathrin in decidual samples in the COVID-19 Group in relation to the NON-COVID-19 Group (*p* = 0.05). The same decrease was also ascertained in samples that tested positive for SARS-CoV-2 RNA in the COVID-19 Group (*p* = 0.013).

This decrease can be explained by the mobilization and utilization of clathrin during viral internalization mechanism. Previous studies have indicated that SARS-CoV-2 can utilize the clathrin-mediated endocytosis pathway for infection of host cells.^22,23^ Thus, the reduced immunoexpression of clathrin in infected patients’ decidua may be indicative of greater reliance on this pathway for viral entry and intracellular trafficking. Furthermore, endosomal entry mechanisms permit effective dissemination of the virus; by engaging with the endocytic pathway, SARS-CoV-2 prevents exposure of its capsid proteins to the immune defenses of the host. This can provide the virus with an advantage by diminishing its detection and the subsequent activation of innate antiviral mechanisms.^22^

The other noteworthy result of the current research, corroborating the hypothesis of viral entrance via the endocytic pathway, was the higher immunoexpression of cathepsin L in COVID-19 Group villus samples when compared with the NON-COVID-19 Group. This result was also complemented by comparing SARS-CoV-2 RNA-positive samples in the COVID-19 Group and the NON-COVID-19 Group.

The upregulation of cathepsin-L expression is consistent with earlier studies that have described its role in different mechanisms involved in viral infections.^24,25^ The current research demonstrates the upregulation of higher levels of this protease in the villous compartment, suggesting a possible role of SARS-CoV-2 in the upregulation of cathepsin-L expression in trophoblast cells. This observation is especially relevant given that these cells do not seem to express essential molecules of the traditional pathways of viral entry, including ACE2 and TMPRSS2.^26,27^

Experiments have demonstrated that following internalization of SARS-CoV-2 via clathrin-mediated endocytosis or other endocytic routes, cathepsin-L acts in the endosomal compartment where it cleaves the Spike protein. This promotes fusion between the viral membrane and the endosomal membrane, thus enabling the release of the viral genetic material into the cytosol.^28,29^

This mechanism is essential for viral infection, particularly in cells that lack the TMPRSS2 pathway. Consequently, the high level of this protease could mean a higher intracellular processing of the virus through the endosomal pathway.

Furthermore, the observation of the same pattern when comparing the NON-COVID-19 Group with placentas from the COVID-19 Group that were confirmed positive for SARS-CoV-2 reinforces this rationale. Gomes et al. (2020) propose that SARS-CoV-2 can influence cathepsin-L expression by alternative splicing of its transcripts, resulting in higher synthesis of this protease and permitting it to replicate intracellularly. This process could be linked to the adaptation of the virus to the placental microenvironment, enabling enhanced efficiency in viral processing through the endocytic pathway.^30^

The current research examined the immunoexpression of proteins permitting SARS-CoV-2 entry into the placenta with the objective of exploring the possible alternative pathways other than the ACE-2 receptor and TMPRSS2 protease. The results revealed that the expression levels of these proteins were comparable in the groups being studied, implicating that the standard pathway may not be the predominant mechanism for placental infection. Conversely, the downregulation of NRP-1 observed in the decidua of the COVID-19 Group, in relation to the viral RNA detection, indicates a potential role for this protein as a secondary pathway of viral entry. Additionally, the downregulation of clathrin with upregulated levels of cathepsin-L reinforces the implication that SARS-CoV-2 can utilize the endocytic pathway for internalization within cells and further intracellular processing for its replication.These findings strengthen the knowledge regarding viral interaction with the placenta and could guide future research on the significance of these alterations in the perspective of maternal SARS-CoV-2 infection.

## Study limitations

Several limitations must be considered in the interpretation of the results. The description of tissue expression, which was conducted on paraffin-embedded samples, is a snapshot record of the process of placental separation. Consequently, the interval between infection and delivery may have influenced viral entry protein expression and thereby precluded an evaluation of the temporal sequence of such changes. Furthermore, because of technical specification constraints, just a part of the paraffin block was submitted for viral RNA recovery and examination, and this may have accounted for the decreased detection of SARS-CoV-2 within some samples. Subsequent studies using complementary approaches, e.g., protein and RNA analyses on fresh specimens or quantitative gene expression techniques, may give an improved understanding of SARS-CoV-2 infection of the placenta.

Immunohistochemical quantification was performed using Image Pro-Plus software. While this method allows reproducible measurement of stained areas, it does not capture subcellular localization or single-cell intensity variations. Future studies employing high-resolution or single-cell image analysis tools could provide more precise localization and quantitative insights.

The significantly lower placental weight observed in the COVID-19 group raises questions about potential clinical consequences. Reduced placental mass has been associated with impaired nutrient transfer, fetal growth restriction, and adverse perinatal outcomes in other contexts. Although our dataset did not systematically collect detailed neonatal anthropometry such as birth weight percentiles or APGAR scores, this finding warrants further investigation to determine whether SARS-CoV-2 infection contributes to functional placental compromise.

All placental samples analyzed in this study were obtained immediately after birth (both term and preterm deliveries). For ethical reasons, and in accordance with institutional and national regulations, it was not possible to prospectively collect placental tissue from ongoing pregnancies in earlier trimesters, as such collection would require invasive procedures that could endanger fetal viability and potentially induce miscarriage. Consequently, the majority of available specimens corresponded to third-trimester gestations, reflecting the natural timing of delivery, and limiting direct comparisons across all gestational ages. Further studies including placentas obtained from earlier trimesters could provide valuable insights into the dynamics of Neuropilin-1 expression during pregnancy. Such investigations may help elucidate whether the observed decrease in Neuropilin-1 levels in the placenta of COVID-19 cases results from consumption or depletion due to viral interaction, or if it reflects other gestational or pathological factors.

## Supplementary Material

Bonferroni supp material-KTIB CLEAN.docx
