# Supplementary material for: Placental infection by SARS-CoV-2: exploring alternative entry pathways
Source: Tissue Barriers. 2025 Nov 30;14(2):2585246. doi: 10.1080/21688370.2025.2585246 (PMC13228962; doi:10.1080/21688370.2025.2585246)
Supplement: Bonferroni supp material-KTIB CLEAN.docx [file KTIB_A_2585246_SM0417.docx]

| **Original *p* value** | **Bonferroni corrected p-value** | **Reject Null Hypothesis** |
| --- | --- | --- |
| 0.301 | 1.0 | False |
| 0.825 | 1.0 | False |
| 0.765 | 1.0 | False |
| 0.648 | 1.0 | False |
| 0.729 | 1.0 | False |
| 0.001 | 0.012 | True |
| 0.078 | 0.9359999999999999 | False |
| 0.05 | 0.6000000000000001 | False |
| 0.286 | 1.0 | False |
| 0.0001 | 0.0012000000000000001 | True |
| 0.059 | 0.708 | False |
| 0.004 | 0.048 | True |
